# Cognitive dysfunction in diabetes – the ‘forgotten’ diabetes complication: a narrative review

**DOI:** 10.1080/02813432.2025.2455136

**Published:** 2025-01-28

**Authors:** Åke Sjöholm, Louise Bennet, Peter M. Nilsson

**Affiliations:** ^a^ Department of Internal Medicine, Division of Endocrinology and Diabetology, Gävle Hospital and University of Gävle, Gävle, Sweden; ^b^ Department of Clinical Sciences, Lund University, Clinical Studies Sweden, Forum South, Skåne University Hospital, Lund, Sweden; ^c^ Department of Clinical Sciences, Lund University, Skåne University Hospital, Malmö, Sweden

**Keywords:** Antidiabetic drugs, cognition, dementia, diabetes, epidemiology

## Abstract

**Background:**

In addition to peripheral neuropathy of various kinds, diabetes can also cause central neuropathy, which among other things can manifest itself as premature cognitive dysfunction, often linked to vascular dysfunction. Although the link between diabetes and cognitive dysfunction was discovered more than 100 years ago and has important clinical implications, this diabetes complication remains relatively unknown. Recent years have seen research that has clarified cerebral insulin resistance and defective insulin signaling as examples of pathogenic factors behind this cognitive impairment in diabetes.

**Method:**

We provide a narrative review of select and contemporary publications with relevance for the interface between diabetes/prediabetes and cognitive function.

**Results:**

Recently published studies show that physical activity can reverse insulin resistance in the brain as well as cognitive impairment and pathological appetite regulation. Pharmacological interventions with, for example, nasal insulin, GLP-1 receptor agonists, SGLT-2 inhibitors, or PPAR-γ agonists have also shown promising results.

**Conclusion:**

Optimization of lifestyle factors (e.g. physical activity), as well as several pharmaceutical agents already in clinical use against diabetes, have shown promising results in improving cognitive function in diabetic patients. An important task for primary health care, where most patients with type 2 diabetes are diagnosed, treated, and followed, is to increase awareness and early detection of cognitive dysfunction in these patients for optimizing risk factor control.

## Introduction

The link between the brain and diabetes has been known since 1855, when the famous French physiologist Claude Bernard discovered that diabetes can be induced by injuring the floor of the fourth ventricle in dogs [1]. Today, we know that specific regions of the CNS have glucose-sensitive neurons [2] and that centrally administered glucose directly impacts blood flow and function of the insulin-producing β-cells in the pancreas [3].

It is also well known that both type 1 diabetes (T1D) and type 2 diabetes (T2D) are associated with an increased risk of severe macroangiopathic complications in the CNS, such as atherosclerosis and ischemic stroke [4]. In addition, diabetes increases the risk of microangiopathic complications, including peripheral neuropathy of various kinds, but is also connected to central neuropathy [5]. The latter is not very well known, even though it was already reported in 1922 that cognitive impairment is increased in patients with diabetes [6].

Extensive clinical experience and population studies show that cognitive dysfunction of various degrees is clearly overrepresented in diabetes, and also in prediabetic states [7–13].

The cognitive impairment in diabetes is not an established or well-defined entity but is increasingly discussed clinically and scientifically.

Cognitive dysfunction has several important diabetes-specific implications. For example, the ability and understanding of the importance of self-care (diet, exercise, etc.) can be negatively affected. The same applies to self-monitoring of glucose as well as adherence to medication prescriptions and understanding of the need to adjust insulin doses. Patients may forget whether they have taken their medication, e.g. insulin, or not, which can cause both hyper- and hypoglycemia and which in turn can further impair cognitive abilities and increase the risk of other diabetic complications.

The purpose of this article is to increase the awareness in primary care of cognitive dysfunction in patients with T2D.

## Etiology and pathogenesis

The mechanisms that put patients with diabetes at increased risk of cognitive dysfunction are not fully understood. Many believe it may be due to cerebral microangiopathy, but this has proven difficult to substantiate. In insulin-treated patients, it has been speculated that recurrent hypoglycemia associated with neuronal cell death would be a cause, but the scientific evidence in support of this is meager [7]. However, a clear correlation with age, diabetes duration, glycemic control, depression and vascular complications has been shown. It is estimated that up to 40% of patients with long-term or less well-controlled diabetes develop varying degrees of cognitive impairment, thus representing a significant comorbidity [7].

The literature is strikingly sparse regarding histopathological changes in the brain of patients with diabetes and cognitive impairment. In diabetic animal models, however, a significant loss of cortical neurons has been observed with increasing age [14] and there is also evidence of cerebral amyloidosis [15].

Although there is consensus that glucose uptake in the CNS is not insulin-regulated, it is important to recognize that insulin is much more than merely a blood glucose-lowering hormone. Insulin is also an anabolic hormone that may have potentially neuroprotective and neurotrophic properties *per se*, and the insulin receptor is expressed in the CNS [16,17].

In recent years, more and more research interest has been focused on insulin signaling and insulin resistance in the CNS, not least their role in cognition [18]. Taken together, these studies indicate that insulin has an important role in the CNS for the control of systemic metabolism, glucose tolerance and body weight, and that obese patients with or without diabetes show insulin resistance also in the brain and that this in turn is linked to cognitive dysfunction and disturbed appetite regulation [16,17].

## Symptoms

Patients themselves seldom express concerns about their cognitive dysfunction, especially at an early stage. Hence, a large proportion may remain undetected until close family members, friends or workplace colleagues react. Suspicion of cognitive impairment usually arises when taking a medical history at clinic visits or when relatives express concern about this. The symptoms do not differ from those of people without diabetes but as shown below, diabetes-specific aspects come into play. The clinical experience is that awareness among caregivers of the strong comorbidity between diabetes and cognitive impairment is often limited, particularly not compared to more well-known diabetes complications.

In Scandinavia, no routine screening of cognitive ability in these patients is applied [19], but both the American Diabetes Association (ADA) and the International Diabetes Federation (IDF) have been recommending this to certain groups for a few years. Testing should be offered to patients over 65 years on an annual basis, according to Standards of Medical Care 2024 from the ADA [20].

Cognitive impairment encompasses a spectrum of conditions, with different degrees of cognitive defects. In early stages of cognitive dysfunction, the condition is reversible.

- *Dementia*. This is the most severe and pronounced degree and, unlike the conditions mentioned below, clearly and adversely affects the patient’s ADL functions. It has been reported that patients with T1D or T2D have a 40–60% increased risk of dementia, especially vascular dementia [7–10].
- *Mild cognitive impairment (MCI)*. This condition involves abnormalities in one or more cognitive domains when tested, but with no or minimal impact on ADL functions. Patients with T2D have a ∼20% increased risk of this condition [7–10], and in addition an increased risk of transition to manifest dementia [21,22].
- *Diabetes-specific cognitive impairment*. This refers to subtle deviations from normal age-related cognitive function, which are not pronounced enough to be classified into the above categories. Patients with T2D have up to half a standard deviation lower cognitive test results than people without diabetes and this affects most cognitive domains (memory, executive functions, etc.) [8–10,23]. The deviation in patients with T1D has been reported to be slightly more pronounced than in T2D [8–10].

## Differential diagnoses

Other forms of dementia, depression and neurodegenerative diseases (e.g. Parkinson’s disease) are overrepresented in diabetes and can cause similar symptoms [8]. Clinically, it is imperative to rule out hypoglycemia, which – especially in the elderly – can produce misleadingly similar symptoms.

Other treatable conditions must also be ruled out, e.g. alcohol over-consumption, vitamin B_12_ deficiency (overrepresented in T1D and a not entirely uncommon side effect of metformin in T2D), thyroid disorders (both hypo- and hyperthyroidism are overrepresented in T1D), hypercalcemia, etc. [8–10].

## Diagnostic evaluation

The investigation process for suspected cognitive impairment in individuals with diabetes does not differ from that in non-diabetic individuals, but the difficulty for clinicians lies in being aware of – and vigilant of – the condition.

- Recurring (at least annual) checks of P-glucose, HbA_1c_, blood pressure, lipids, vitamin B_12_, folate, blood levels of phosphatidyletanolamine (B-PEth) and thyroid profile.
- In insulin- or sulfonylurea-treated patients, continuous glucose monitoring (CGM) of glycemic fluctuations to identify frequency and degree of hypoglycemia and glycemic variability.
- Cognitive testing might be considered, as discussed in recent guidelines from the ADA [20].
- Consideration of brain imaging like CT scans as part of the basic evaluation in early stages of dementia.
- Testing with a self-report instrument for depression (e.g. Hospital Anxiety and Depression Scale [24]).

## Treatment

In addition to glucose-lowering therapy, it has previously been shown that lowering blood pressure in systolic hypertension [25] reduces the risk of developing dementia. In contrast, the benefits of lipid lowering in these patients are more ambiguous [26].

## Lifestyle intervention

As in all types of diabetes, optimized lifestyle factors form the basis of treatment. All forms of physical activity as well as smoking cessation should be actively encouraged. Dietary advice, physical activity and cognitive training have been shown to slow down cognitive decline in older individuals [27,28]. Interestingly, it has been recently reported that physical activity improves both insulin sensitivity in the CNS and cognitive abilities in humans, in parallel with reduced feelings of hunger [29].

## Pharmacological treatment

In addition to the drugs already available for cognitive impairment, such as choline esterase inhibitors for prevention of Alzheimer dementia [30], the choice of antidiabetic drugs is a daunting task with special challenges in this vulnerable patient group. International guidelines emphasize the importance of individualized treatment, simple treatment regimens and avoidance of drugs that may cause hypoglycemia [31].

Care must be taken to avoid lowering HbA_1c_ too much with drugs that can cause hypoglycemia (i.e. insulin and sulfonylureas) in the very elderly and frail patients, especially in those with low BMI and fluctuating glycemia [31].

In T2D management, there are currently several drug classes that do not cause hypoglycemia (if they are not combined with insulin or sulfonylureas), e.g. metformin, DPP-4 inhibitors, GLP-1R agonists, SGLT-2 inhibitors and the PPAR-γ agonist pioglitazone [32].

**Figure 1.**
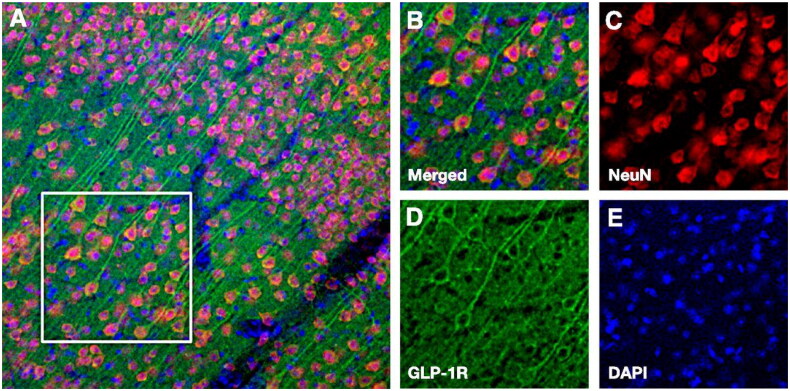
The GLP-1 receptor is expressed in cortical neurons. (A) Low magnification image of GLP-1R expression in mouse cerebral cortex. (B) High magnification image corresponding to the white box in panel A. Split channel images show immunoreactivity for the neuronal cell marker NeuN (neuronal nuclear protein) (C, in red), the GLP-1 receptor (D, in green) and the nuclear marker DAPI (4′,6-diamidino-2-phenylindole) (E, in blue). From reference [34] with permission from the American Diabetes Association.

GLP-1 (glucagon-like peptide-1) and its receptor agonists/analogues appear interesting in this context for several reasons. Plasma levels of GLP-1 are reduced in T2D, high plasma levels of GLP-1 correlate with good cognition [12] and GLP-1 has been reported to be produced in the CNS [33] which also expresses the GLP-1 receptor in different regions [34] (see Figure 1). In the REWIND study, a reduction in cognitive impairment was noted in the patients with T2D who received treatment with the GLP-1 receptor agonist dulaglutide compared to placebo [35].

However, in studies with the DPP-4 inhibitor linagliptin, which does not cross the blood–brain barrier but moderately increases plasma levels of GLP-1 [34], no change in cognitive functions was noted compared to sulfonylurea in patients with T2D [36,37].

Systemic insulin treatment has not been convincingly shown to affect the level of cognition and has obvious disadvantages, such as the risk of hypoglycemia and weight gain, which by themselves can impair cognitive functions. Intranasal insulin, in contrast, has been shown in some studies to both improve cognition and to lower insulin resistance in the CNS, but without systemic effects on glycemia or body weight [17,18,38].

In a large prospective observational study with >550,000 elderly patients with T2D [39], it was shown that treatment with sulfonylureas was associated with an increased risk of dementia compared to metformin, and that treatment with PPAR-γ agonists (thiazolidinediones) was associated with a reduced risk of dementia. In another recently published study of >112,000 patients (50 years and older) with T2D, it was shown that patients on metformin therapy had a 20% lower incidence of dementia over a five-year period, compared to patients on sulfonylurea therapy [40].

Several clinical trials, reviews and meta-analyses with various antidiabetic drugs against cognitive impairment and early dementia – with or without diabetes – have been published [41–48] or are ongoing (Table 1), according to www.clinicaltrials.gov.

**Table 1.** Published and ongoing randomized clinical trials regarding effects of treatment for the prevention of impairment of brain functions in diabetic patients.

Study	Target group	Intervention	Outcome	Comment
ACCORD-MIND ACCORDION [49]	T2D	Intensive vs. standard glucose control	No differences in cognition or dementia	Part of a larger study that was prematurely terminated
CAROLINA-Cognition [36]	T2D	Linagliptin vs. glimepiride	No difference in cognitive decline	Interpretation difficult due to lack of placebo arm
REWIND [35]	T2D	Dulaglutide vs. placebo	Dulaglutide better than placebo in preventing cognitive decline	A post hoc analysis of a larger study, hypothesis-generating
EVOKE https://clinicaltrials.gov/ct2/show/NCT04777396	T2D	Semaglutide vs. placebo	Ongoing	
DRINN https://beta.clinicaltrials.gov/study/NCT02847403	Pre-diabetes	Exenatide vs. placebo	Ended 2021	Not published
MET-FINGER [50] https://clinicaltrials.gov/study/NCT05109169	Mixed	Metformin + lifestyle vs. placebo	Ongoing	

## Perspectives for primary care

In diabetes patients, clinical risk factors for cognitive decline include age >60 years, long duration of diabetes, poor glycemic control, poor blood pressure control, high fasting glucose levels, recurrent hypoglycemic episodes and severe insulin resistance. In addition, adverse social factors and low educational level can add to the risk. Several comorbidities can increase the risk of cognitive decline including obesity, history of cardiovascular disease and proteinuria [51]. A systematic review and meta-analysis reports that in people with diabetes, depression is associated with worse cognition and higher dementia risk [52]. The potential mitigating effect of antidepressant treatment remains unclear, as divergent results of such intervention were reported in that meta-analysis [52].

Specialists in family medicine regularly monitor T2D patients regarding cardiovascular and metabolic risk factors, but it is still a challenge in primary health care to identify people with T2D at risk for cognitive dysfunction. Increased awareness, a thorough medical history including targeted questions to the patient and her/his relatives addressing depression, cognitive and memory function, and a health exam are of importance.

Liberal screening for cognitive dysfunction using, for instance, the MoCA test [53] or executive function tests, could be valuable at an early stage identifying patients at risk. Effective interventions in diet, medications, biochemical exposures, psychological condition, pre-existing disease and lifestyle may decrease or postpone new incidence of dementia [54].

Treatment with antihypertensives acting on the renin–angiotensin–aldosterone system (RAAS) has been associated with improved cognitive function in patients with diabetes in terms of executive abilities, processing speed and verbal memory compared to those treated with other antihypertensives [55].

Newer glucose-lowering drugs were associated with a decreased risk of all-cause dementia in people with T2D. Because of the observational nature and significant heterogeneity between studies, the results should be interpreted with caution. Further research is warranted to confirm such findings [56]. In a three month randomized controlled trial, the GLP-1R agonist liraglutide improved scores in all cognitive function tests including memory and attention compared to the control group [57], a similar effect by liraglutide seen in non-diabetic obese patients [58].

The protective effects on cognitive dysfunction observed with of pioglitazone [59] warrant further investigation in randomized trials.

Long-term follow-up randomized controlled trials with intensive lifestyle intervention show reduced risk of cognitive complaints in those without cognitive dysfunction at baseline [60,61]. This illustrates the importance of continuous risk factor assessment in primary care supporting and motivating patients with T2D to have and keep healthy habits since it impacts cognitive health and function [49,50].

## Conclusions

A large proportion of elderly patients with diabetes are at risk for cognitive dysfunction and many are probably still undiagnosed. This can adversely influence their treatment compliance, ability to reach treatment targets and increase their risk of future complications, morbidity and mortality. Future clinical trials with long-term follow-up are needed to identify glucose lowering medications that prevent cognitive dysfunction and prevent or postpone the development of dementia in people with T2D. Of special importance is to encourage primary care physicians to look for signs of cognitive dysfunction or pre-dementia in their T2D patients, and to tailor both lifestyle interventions and drug treatment to the individual needs of these patients – a work still in need to be more developed.
